# Secretion of Pertussis Toxin from *Bordetella pertussis*

**DOI:** 10.3390/toxins13080574

**Published:** 2021-08-18

**Authors:** Drusilla L. Burns

**Affiliations:** Center for Biologics Evaluation and Research, U.S. Food and Drug Administration, Silver Spring, MD 20993, USA; drusilla.burns@fda.hhs.gov

**Keywords:** toxin secretion, type IV secretion, pertussis toxin

## Abstract

Production and secretion of pertussis toxin (PT) is essential for the virulence of *Bordetella pertussis*. Due to the large oligomeric structure of PT, transport of the toxin across bacterial membrane barriers represents a significant hurdle that the bacteria must overcome in order to maintain pathogenicity. During the secretion process, PT undergoes a two-step transport process. The first step involves transport of the individual polypeptide chains of PT across the inner membrane utilizing a generalized secretion pathway, most likely the bacterial Sec system. The second step involves the use of a specialized apparatus to transport the toxin across the outer membrane of the bacterial cell. This apparatus, which has been termed the Ptl transporter and which is unique to the PT secretion pathway, is a member of the type IV family of bacterial transporters. Here, the current understanding of the PT secretion process is reviewed including a description of the Ptl proteins that assemble to form the transporter, the general structure of type IV transporters, the known similarities and differences between canonical type IV substrate transport and Ptl-mediated transport of PT, as well as the known sequence of events in the assembly and secretion of PT.

## 1. Introduction

Pertussis toxin (PT), similar to other bacterial protein toxins, is a highly evolved protein that must complete multiple tasks in order to attain its ultimate goal of intoxicating mammalian cells. During the course of the toxin’s lifespan, the toxin must correctly fold and assemble, cross two bacterial cell membranes, find its way to its target eukaryotic cell, bind and enter that cell, correctly traffic through the eukaryotic cell to its site of action, and finally, enzymatically modify its G protein substrate resulting in intoxication of that cell. This review will focus on the molecular events occurring early in that process, i.e., toxin assembly and secretion from *Bordetella pertussis.*

PT is a typical A-B bacterial protein toxin in that it is composed of an enzymatically active A component and a binding B component. However, unlike the simple monomeric structure of a number of A-B toxins, PT is multimeric, composed of the catalytically active S1 subunit and five subunits (one copy each of subunits S2, S3, and S5 as well as two copies of subunit S4) that make up the B oligomer which is the receptor binding component of the toxin.

The complexity of this structure and the size of the assembled holotoxin add to the challenge of transporting the toxin across the bacterial cell membranes. Each PT subunit is synthesized with its own signal sequence [1,2], suggesting that the individual subunits are individually transported across the inner membrane using the bacterial Sec system or a Sec-like system.

The outer membrane presents the next barrier that the toxin must cross. To accomplish this task, *B. pertussis* utilizes a specialized transport system, known as the Ptl system composed of nine different proteins (PtlA-PtlI) [3,4]. Each of the Ptl proteins is essential for protein secretion [5]. The *ptl* genes encoding the transporter are part of the *ptx-ptl* operon comprising both the *ptx* genes, which encode the toxin subunits, and the *ptl* genes. The *ptl* genes are located directly downstream from the *ptx* genes [4]. Thus, the genes encoding both the toxin subunits and the toxin secretion apparatus are coordinately expressed. The Ptl system belongs to the type IV family of bacterial transporters [6,7] that are found in a number of Gram-negative bacterial species as well as some Gram-positive bacteria.

## 2. Type IV Transporters

Members of the family of type IV transporters mediate the transport of proteins and/or DNA across bacterial membranes. While having evolved from a common ancestor, different type IV transporters carry out diverse functions including bacterial DNA conjugation (transfer of DNA), DNA transport into eukaryotic cells (transfer of nucleoprotein complexes), and protein transport including toxin secretion (protein transfer) [8,9]. Recently, type IV transporters have also shown to be involved in interbacterial warfare leading to bacterial killing [10]. A number of Gram-negative bacterial species are known to produce type IV transporters including non-pathogenic species such as *E. coli* and pathogenic species such as *B. pertussis*, *Agrobacterium tumefaciens*, *Brucella* spp., *Bartonella* spp., *Helicobacter pylori*, and *Legionella pneumophila* [11,12,13,14,15,16]. Type IV transporters have also been described for Gram-positive organisms [11].

The payloads transported by type IV systems can be key to bacterial survival, especially for pathogenic bacteria that use these systems to secrete essential virulence factors into the extracellular milieu or to transport virulence factors directly into mammalian host cells. The diversity of type IV transporter substrates is great, ranging from DNA to proteins, as is the diversity in the number of substrates transported by any given system. The Ptl system of *B. pertussis* is known to have only a single protein substrate, PT [6]. The *H. pylori* system also has only one known protein substrate, CagA, an oncoprotein that alters stomach cells in a way that can lead to gastric cancer [17,18]. In contrast, the type IV dot/icm system of *L. pneumophila*, which is essential for virulence of this organism, is known to transport over 300 different effector proteins into target mammalian cells [15]. Unlike these protein transporters, other type IV transporters are involved in the conjugal transfer of DNA (e.g., the type IV systems of pKM101 and R388 plasmids) or the oncogenic transfer of DNA from bacteria to eukaryotic cells by the type IV transporter of *A. tumefaciens* [19,20].

**Figure 1:**
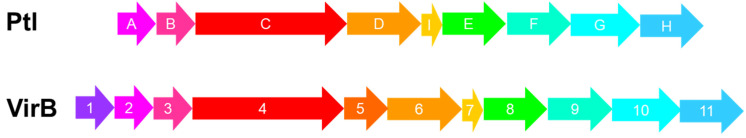
Genes encoding the Ptl transporter of *B. pertussis* (**top**) and the VirB transporter of *A. tumefaciens* (**bottom**). Arrows of the same color represent homologous genes [4].

The prototypical type IV system, as exemplified by the VirB system of *A. tumefaciens*, consists of 11 VirB proteins, VirB1–VirB11. The genetic organization of the *A. tumefaciens virB* operon is shown in Figure 1. The *ptl* genes of *B.* pertussis (Figure 1) display significant homology to the *virB* genes of *A. tumefaciens* [4]. Some type IV systems, such as the dot/icm system of *L. pneumophila*, are more complex and contain a subset of the VirB proteins as well as a number of additional proteins [21].

Over the years, many studies have helped elucidate the specific roles of the different VirB proteins. VirB3, VirB6, VirB7, VirB8, VirB9, and VirB10 are thought to be the major structural components of the transporter [9,19,22,23,24]. VirB2 has been identified as a major pilin subunit that multimerizes to form the pilus that is associated with certain type IV transporters such as the *A. tumefaciens* VirB transporter [25,26]. The VirB2 pilin of *A. tumefaciens* has an unusual cyclized structure [27,28]. VirB5 is a minor pilin subunit that has been localized to the tip of the pilus and is believed to be involved in host cell recognition [29,30]. VirB4 and VirB11 are ATPases that power transport and pilus biogenesis [31,32,33]. VirB1 is a transglycosylase that is thought to lyse the peptidoglycan layer [34,35]. The Ptl system contains homologues to each of these proteins with the exceptions of the transglycosylase VirB1 and the minor pilin adhesin VirB5.

**Figure 2:**
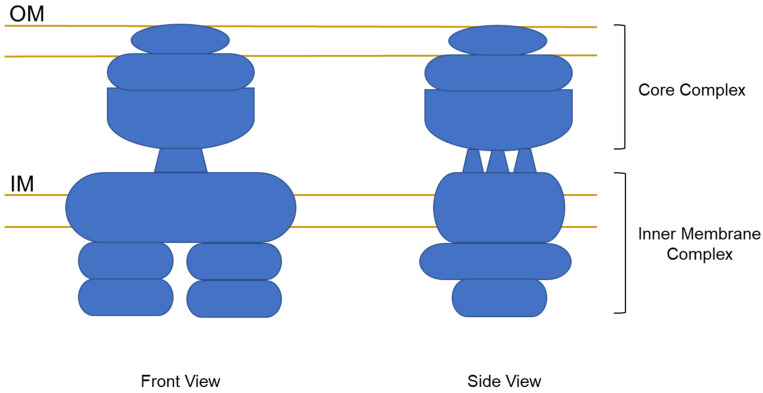
Schematic drawing of the structure of a type IV transporter complex consisting of homologs of VirB3-VirB10. The drawing is adapted from Low et al. [19].

A substructure of the type IV transporter of the *E. coli* R388 conjugative plasmid comprising VirB3–VirB10 homologues has been elucidated using electron microscopy [19]. While this substructure does not provide a complete picture of the entire transporter, the substructure contains most of the proteins that are thought to make up the main scaffolding of the transporter. The VirB3–VirB10 complex is quite large and spans both the inner and outer membranes. A schematic drawing of that structure is shown in Figure 2. Given the homology between the Ptl proteins and corresponding VirB proteins, this structure is likely to generally reflect that of the Ptl transporter.

The VirB3-VirB10 structure indicates that the transporter is composed of an outer membrane core complex and an inner membrane complex connected by a central stalk. The inner membrane complex of the transporter is believed to be composed of VirB3, VirB4, VirB6, VirB8, and the N-terminus of VirB10 [9,19] although specific subunit interactions and detailed architecture have yet to be determined. The structure of the outer membrane core complex has been better described based on electron microscopic and x-ray crystallographic analysis of the outer membrane core complex of the type IV transporter of the conjugative KM101 plasmid [22]. From this work, VirB10 has been localized to the inner wall of the structure with VirB7 and VirB9 wrapping around it to form an outer wall of the structure.

Support for similarities between the outer membrane core complex of the Ptl system and that of other type IV transporters comes from a study of the functionality of chimeric type IV systems composed of the inner membrane complex from the pKM101 Tra conjugation system and Tra-Ptl chimeras of the outer membrane core complex [36]. These investigators found that the Tra::Ptl chimera supported conjugative DNA transfer of the pKM101 substrate, although to a lower level than the native system. However, even this lower level of transfer was notable since the Tra system transfers a DNA substrate, whereas the Ptl system transfers a large protein toxin.

The type IV transporter structure depicted in Figure 2 does not include the pilus structure, believed to be composed of a major pilus subunit (VirB2) and a minor pilus tip adhesin (VirB5), that has been associated with at least certain type IV transporters, including the VirB transporter of *A. tumefaciens* [25]. The Ptl system contains a protein, PtlA, that is homologous to VirB2, the major pilin subunit [4]. However, despite attempts to visualize a Ptl pilus structure, no such structure has been reported. The lack of an observable pilus structure could be due to technical issues or alternatively, the Ptl system may lack such a pilus structure. This latter possibility may be supported by the finding that the *A. tumefaciens* VirB pilus is not an absolute requirement for substrate transport [37,38] since certain mutations in VirB10 can block pilus biogenesis, but not substrate transfer. Moreover, a chimeric Tra::Ptl system, composed of the inner membrane complex of the *E. coli* pKM101 Tra system that mediates conjugative DNA transfer between bacteria and the outer membrane core complex of the Ptl transporter, fails to elaborate detectable pili but can support conjugative DNA transfer [36]. Trokter et al. [9] have suggested that type IV systems may exist in two states, one that is secretion-competent and the other that is pilus-biogenesis competent, extending either a short or a long pilus, respectively. In such a scenario, the Ptl system might represent a mostly secretion-competent state in which only a short, not easily observable, pilus is elaborated. The Ptl system is also missing a homologue of the minor pilin tip protein, VirB5, that is believed to be involved in host cell recognition, possibly as an adhesin. As an adhesin, VirB5 could play a role in attachment of the transport apparatus to the cell that receives the substrate payload. The Ptl system would not be expected to need such a protein, since PT is secreted into the extracellular milieu rather than being directly introduced into its target mammalian cell. Perhaps the B oligomer of PT, the toxin’s binding component, serves as a substitute for VirB5 since it functions to bind PT to receptors on the mammalian cell and therefore attach the toxin to its target cell. Of note, however, none of the B oligomer subunits have any significant homology to VirB5.

The Ptl system also lacks a VirB1 homologue. While VirB1 has been identified as a transglycosylase [34,35], the exact role that VirB1 plays during the transport process is not entirely clear. VirB1 is necessary for pilus biogenesis [25], but is not essential for the translocation channel since VirB1 mutants are attenuated, but not completely avirulent [11,39]. This finding might explain why a VirB1 homologue might not be needed for PT transport since the Ptl system has not been associated with a pilus structure. Of note, however, Rambow-Larson and Weiss reported that PtlE has peptidoglycanase activity and suggested that PtlE might perform the function associated with VirB1 and its homologues [40].

The sequence of events that occur as a substrate is transported through a type IV system is not well understood. Using a novel cross-linking technique, Cascales and Christie were able to glean insight into the events that occur during transport of the DNA substrate, known as T-DNA, through the VirB transporter of *A. tumefaciens* [41]. They found that the first VirB protein to interact with the substrate is VirB11. As the substrate progresses through the transporter, this interaction is followed by interaction with VirB6 and VirB8, followed by interaction with VirB2, the major pilin subunit, and VirB9. Due to the direct interaction of these VirB proteins with the T-DNA substrate, Cascales and Christie postulated that these proteins correspond to channel subunits of the secretory apparatus [41]. These results are consistent with the known structure of VirB transporters [21]. If the T-DNA enters the transporter at the inner membrane, VirB11 would be expected to be one of the first VirB proteins to be encountered since that protein is thought to be located on the cytoplasmic side of the inner membrane. VirB6 and VirB8 have been identified as inner membrane complex components, and therefore would next be accessible to the substrate. Only after the substrate continues through the transporter would it be accessible to those VirB proteins located in the outer core complex such as VirB9. Despite these advances in our knowledge of the interaction of the T-DNA substrate with its VirB transporter, very little is known about how PT might interact with the Ptl transporter and the events that occur during toxin secretion including both the similarities and differences that may exist between the events occurring during PT transport and those occurring during T-DNA transport.

## 3. Sequence of Events in the Assembly and Secretion of PT

As mentioned above, each PT subunit is synthesized with its own signal sequence suggesting that the individual subunits are transported across the inner membrane by a Ptl-independent pathway such as the Sec system. Once in the periplasm, PT subunits would then gain access to the Ptl system. Precedent exists for a two-step mechanism for type IV transport since certain proteins that are components of *A. tumefaciens* type IV transporter nucleoprotein substrates are transported through the inner membrane by a VirB-independent mechanism [42]. Indeed, a possible two-step transport mechanism has been suggested [9], with the protein substrate first being transported across the inner membrane and then gaining access to and entering the outer membrane complex of the transporter in the periplasm.

**Figure 3:**
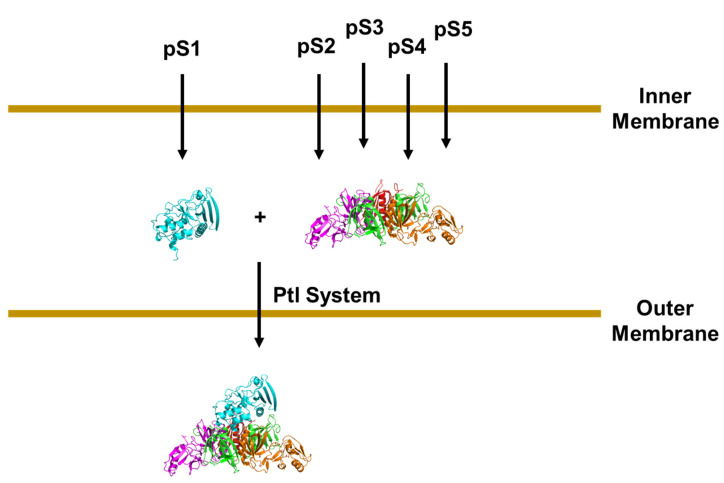
Schematic depiction of the secretion of PT from *B. pertussis*. Individual polypeptide chains are synthesized with a signal sequence (pS1–pS5). The individual chains are then transported across the inner membrane by a Ptl-independent pathway such as the Sec pathway and their signal sequences are cleaved. The S1 subunit and the subunits comprising the B oligomer (S2–S5) then assemble before the toxin is released from the bacterial cell.

The question arises as to where PT assembly takes place. Are PT subunits independently exported by the Ptl system with assembly occurring in the extracellular milieu after secretion or does assembly take place before release of the toxin from the bacterial cell? Several studies suggest the latter scenario [43,44]. Farizo et al. [43] reported that the S1 subunit was not efficiently secreted in a strain of *B. pertussis* expressing the gene encoding the S1 subunit but not the genes encoding the B oligomer subunits even though the strain had an intact Ptl system. Likewise, in a strain of *B. pertussis* with an intact Ptl system and expressing the genes encoding the B oligomer subunits but not the gene encoding S1, the B oligomer was not secreted. Antoine and Locht [45] had earlier reported on a study in which they found that the B oligomer could be secreted from *B. pertussis* in the absence of a stable form of S1. However, they noted that significant amounts of B oligomer remained cell associated in those mutant strains of *B. pertussis* that did not produce a stable form of S1 as compared to that observed with the wild-type strain. Taken together, the data suggest a series of events as depicted in Figure 3 with PT assembling before its efficient release from the bacterial cell.

The S1 subunit was noted to have a C-terminal motif that resembles a motif that directs bacterial proteins to the outer membrane [46]. Moreover, in a strain of *B. pertussis* that does not produce the B oligomer, the S1 subunit was shown to have triton-X 100 insolubility that is characteristic of bacterial outer membrane proteins [46]. These results suggest that, after synthesis but before secretion, the S1 subunit may localize to the bacterial outer membrane. Thus, the outer membrane may serve as a nucleation site for assembly of the S1 subunit with the B oligomer. The question remains as to whether the S1 subunit is localized to the periplasmic or extracellular side of the outer membrane, and thus the exact site of assembly of the holotoxin remains unknown.

## 4. Conclusions and Future Questions

The discovery over 20 years ago that PT utilizes a type IV transporter to cross bacterial membrane barriers represented a substantial advance in our understanding of PT secretion. The finding was especially intriguing since many type IV transporters represent DNA conjugal systems rather than toxin or effector transporters, raising the question of how this toxin transporter evolved from its DNA transporter relatives. While the secretion pathway for PT and the type IV apparatus used for its transport are now better understood, many important questions remain. What is the sequence of events in the transport process? What directs the PT subunits to the transporter? How and where do the PT subunits gain access to the transporter? The crystal structure of PT indicates that the holotoxin molecule has dimensions of approximately 60Å × 60Å × 100Å [47]. How does this large protein fit through the transporter? Does PT assemble in the periplasm or on the surface of the cell while still interacting with transporter components? Do the individual subunits need to unfold during the transport process?

While substantial progress has been made in understanding the structure of the type IV family of transporters, the structures of many type IV substrates differ dramatically from the oligomeric protein structure of PT. Thus, questions remain as to how the Ptl system might differ from that of prototypical type IV transporters in order to accommodate dramatically different transport substrates. Clearly, more work is needed in order to get a detailed understanding of the PT secretion process.

## Data Availability

Not applicable.
